# Can Ki-67 serve as a suitable marker to indicate the necessity of staging diagnostics in cases of low-risk breast cancer?

**DOI:** 10.1007/s00404-024-07753-2

**Published:** 2024-10-30

**Authors:** Lisa Jung, Sarah Isabelle Huwer, Peter Jungmann, Markus Medl, Florin-Andrei Taran, Jakob Neubauer, Carolin Wilpert, Ingolf Juhasz-Böss, Carolin Müller

**Affiliations:** 1 Department of Obstetrics and Gynecology at the Medical Center, University of Freiburg, Hugstetterstr. 55, 79106 Freiburg, Germany; 2 Department of Radiology, University Medical Center Freiburg, University of Freiburg, Freiburg, Germany; 3 Department of Obstetrics & Gynecology, Saarland University Medical Center, University of Saarland, Homburg, Germany; 4 Department of Anesthesiology, Outcomes Research Consortium, Cleveland Clinic, Cleveland, OH USA

**Keywords:** Breast cancer, Ki-67, Staging, Diagnostics, Low risk, Distant metastases

## Abstract

**Background:**

For many years, staging tests have not been routinely employed for low-risk early breast cancer (EBC). However, the role of Ki-67 in determining the need for staging tests in low-risk EBC remains unclear. Our study aimed to assess the number and types of staging diagnostics, additional imaging, false-positive results, and rate of distant metastases in low-risk EBC with low and high Ki-67 (< / ≥ 25%).

**Methods:**

This is a retrospective, single institution cohort study. All patients with newly diagnosed low-risk breast cancer at the University Medical Center in Freiburg in 2017 and 2021 were included. Low-risk was defined as clinical tumor stage T1/2, node negative (N0), hormone receptor positive, HER2 negative, asymptomatic EBC. Information on demographics, clinical and pathological characteristics, as well as number and type of performed staging diagnostics was obtained. Rate and type of additional imaging or follow-up diagnostics due to suspicious findings was analyzed. The patients were divided into two groups (Ki-67 < and ≥ 25%) and rates of distant metastases, performed staging diagnostics and false positive rates were compared.

**Results:**

A total of 189 patients with low-risk EBC were identified, with 54% (n = 102) having Ki-67 < 25% and 46% (n = 87) having Ki-67 ≥ 25%. Risk for distant metastases was 0% in Ki-67 < 25% and 1.1% in patients with Ki-67 ≥ 25% (p = 0.46). Due to suspicious findings in the initial staging diagnostic, additional imaging was required for 11.8% (n = 12) of patients with Ki-67 < 25% compared to 19.5% (n = 17) of patients with Ki-67 ≥ 25% (p = 0.16). False positive rates did not differ significantly between the two groups (7.6% in Ki-67 < 25% vs. 9.8% in Ki-67 ≥ 25%; p = 0.55).

**Conclusion:**

Distant metastases are rare in low-risk EBC. All in all, staging diagnostics should not be routinely employed in this patient population. Only patients with high Ki-67 developed distant metastases. In these cases, staging diagnostics may be discussed with the patient.

## Introduction

Breast cancer is the most common malignancy in women worldwide [1]. Identifying distant metastasis is important for treatment planning and evaluation of prognosis [2]. However, the incidence of distant metastases at initial presentation is generally low, around 4% [3]. Particularly patients with early breast cancer (EBC) and low risk factors are unlikely to suffer distant metastases [4]. Hormone receptor-positive, HER2 negative, and node-negative breast cancer with low tumor stage, indicate a favorable prognosis [5].

Breast cancer primarily spreads via the lymphatic system (locally), and less frequently through the bloodstream [6]. If distant metastases occur, the predominant sites are bones, lungs, liver, and brain [6]. Until recent years it was part of the clinical routine to perform additional imaging as staging diagnostics for every patient with newly diagnosed breast cancer. Common procedures included computed tomography (CT) of the chest and abdomen, along with bone scintigraphy. In the past, instead of CT, ultrasound examinations of the upper abdomen and chest X-rays were used [3]. Additional imaging procedures for EBC were performed heterogeneously within different healthcare systems [3]. However, due to the low incidence of metastases in EBC patients, there was a high rate of false positive findings if all patients receive staging diagnostics [3]. Low-risk EBC patients underwent an average up to three radiological examinations, despite the very low risk (0.5%) of detecting distant metastases [7]. Therefore, current national and international guidelines do not recommend routine staging diagnostics in EBC with low risk and no symptoms for distant metastases [2, 8].

The proliferation marker Ki-67 is one of the indicators used to differentiate between high-risk and low-risk patients as it was shown previously that a high Ki-67 is associated with lower disease-free and overall survival (OS) [9, 10]. A meta-analysis involving approximately 64,000 patients identified a Ki-67 threshold of ≥ 25, which was linked to poorer OS (HR: 2.05, 95% CI 1.66–2.53, P < 0.00001) [10]. Therefore, Ki-67 is listed as a prognostic marker in national and international guidelines [11, 12]. These guidelines further recommend staging diagnostics in EBC patients with either positive lymph nodes (N +), large tumor size (> T2), clinical signs / symptoms of metastases or aggressive biology [11, 12]. However, they do not specify the necessity of staging for low-risk EBC with high Ki-67 levels. [11, 12]. Consequently, it remains unclear whether staging should be performed in exceptional cases of low-risk carcinoma. This is particularly relevant for patients with EBC and high Ki-67 (≥ 25%), as Ki-67 has been identified as an independent prognostic parameter for OS [10]. The aim of this study was to evaluate whether Ki-67 is an appropriate marker for determining the necessity of staging diagnostics in cases of low-risk EBC. We hypothesized that patients with EBC and high Ki-67 (≥ 25%) have a higher incidence of distant metastases, and therefore lower rates of false positive findings in the initial staging diagnostics compared to patients with low Ki-67 (< 25%).

## Methods

**Fig. 1:**
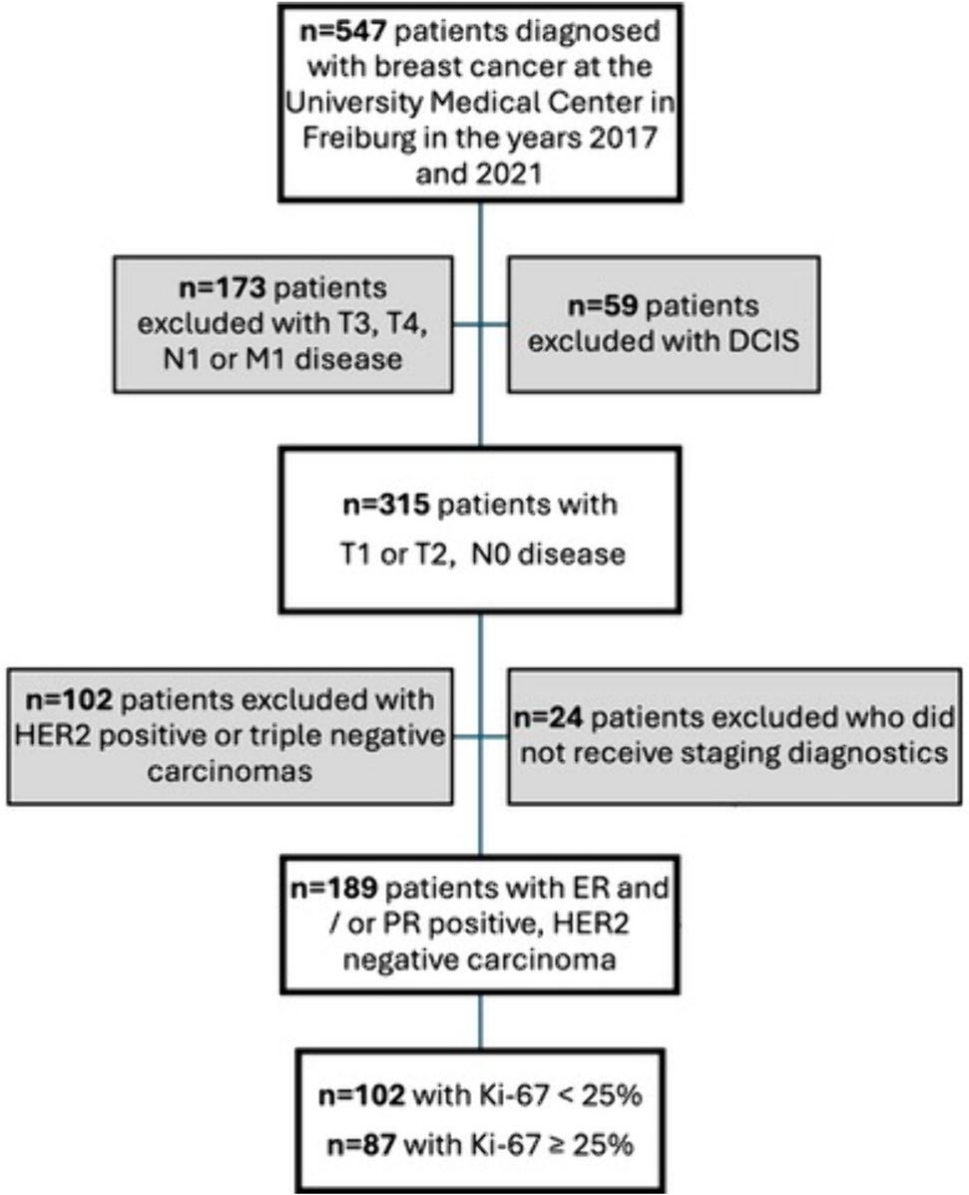
Patient selection based on inclusion and exclusion criteria

This is a retrospective, single institution cohort study, which was approved by the Institutional Review Board (24–1197-S1-retro). All patients with newly diagnosed low-risk breast cancer at the University Medical Center in Freiburg in the years 2017 and 2021 were enrolled. Low risk was defined as clinical tumor stage T1 or T2, node negative (N0), grading G1-3 (Elston and Ellis [13]), HER2 negative, estrogen receptor (ER) and/or progesterone receptor (PR) positive. Only patients who underwent initial staging diagnostics at diagnosis and exhibited no symptoms of distant metastases were included. Exclusion criteria were node positive disease (N +), symptoms or proof of distant metastases (M1) and HER2 positive, as well as triple negative tumors. Patient selection is demonstrated in Fig. 1. Information on demographics, diagnostics, clinicopathological data and treatment data were retrieved from the hospital’s digital documentation system (Prometheus) and the tumor registry. The diagnostic imaging conducted following the breast cancer diagnosis was regarded as initial staging diagnostics. All suspicious findings were documented. The resulting additional imaging for clarification prior to therapy, as well as necessary follow-up diagnostics (e.g., after 3 or 6 months in the event of abnormal findings) were documented. Patients were divided into two cohorts: 1) Ki-67 < 25% and 2) Ki-67 ≥ 25%. Ki-67 was assessed according to the recommendations of the “International Ki-67 in Breast Cancer Working Group” and national practice guidelines [14, 15]. A standardized staining protocol with internal and external quality control was used. Evaluation of Ki-67 was carried out in at least 3 visual fields at high magnification (400x). The relation of Ki-67 positive tumor cells to the total number of tumor cells was used to determine the percentage of Ki-67.

The occurrence of distant metastases, type of initial staging diagnostics, along with the subsequent additional diagnostics in case of suspicious findings, and rate of false positive findings were considered separately in both groups (Ki-67 < 25% and Ki-67 ≥ 25%).

For the statistical analyses, Microsoft Excel 2010 (Microsoft, Redmond, WA, USA) and SPSS 28.0 (IBM, Armonk, USA) was used. Normal distribution in quantitative parameters was tested with the Kolmogorov–Smirnov test. Consequently, parameters are presented as mean with standard deviation (if normally distributed) or median with minimum and maximum (if not normally distributed). Qualitative parameters are presented as absolute frequencies and percentages. Occurrence of distant metastases, number of additional imaging (due to suspicious findings in the initial staging diagnostic) and false positive rates were calculated for the two cohorts (Ki-67 < 25% and Ki-67 ≥ 25%). Furthermore, both cohorts were compared using Fisher’s exact test. P < 0.05 was considered statistically significant.

## Results

A total of n = 547 patients was diagnosed with breast cancer at the University Medical Center in Freiburg in 2017 and 2021. Of these patients, n = 189 patients met all in- and exclusion criteria, see Fig. 1. Patients’ characteristics are presented in Table 1. The mean patient age was 60 years (± 12.5). 17.5% (n = 33) had clinical stage T1 and 82.5% (n = 156) T2. All patients were node negative (N0). Most tumors were moderately differentiated (G2: 72.5%, n = 137) and all patients were hormone receptor positive, as well as HER2 negative. Median Ki-67 in all patients was 23.9% (min 1%, max 80%). Regarding the two cohorts, a total of 54% (n = 102) had Ki-67 < 25% and 46% (n = 87) had Ki-67 ≥ 25%.

**Table 1:** Patient characteristics

Patient characteristics	
Age	60 (± 12.5)
Stage	
T1	156 (82.5%)
T2	33 (17.5%)
Nodal status	
N0	189 (100%)
Metastatic disease	
M0	188 (99.5%)
M1	1 (0.5%)
Grading	
G1	31 (16.4%)
G2	137 (72.5%)
G3	21 (11.1%)
Estrogen receptor (ER)	
Positive	186 (98.4%)
Negative	3 (1.6%)
Progesterone receptor (PR)	
Positive	178 (94.2%)
Negative	11 (5.8%)
HER2 receptor	
Positive	0
Negative	189 (100%)
Total	189 (100%)

Table 2 shows the initial staging diagnostics for all patients. 40.2% (n = 76) received chest X-ray and upper abdominal sonography, whereas 29.6% (n = 56) had CT thorax and abdomen as initial staging diagnostic. Bone scintigraphy was performed in 64.6% (n = 122) of cases.

**Table 2:** Initial staging diagnostics of all (n = 189) patients

Initial staging diagnostics for all patients		
Chest x-ray	Yes	76 (40.2%)
	No	113 (59.8%)
Upper abdominal sonography	Yes	76 (40.2%)
	No	113 (59.8%)
Bone scintigraphy	Yes	122 (64.6%)
	No	67 (35.4%)
CT thorax and abdomen	Yes	56 (29.6%)
	No	133 (70.4%)
PET-CT	Yes	5 (2.6%)
	No	184 (97.4%)
CT thorax**	Yes	5 (2.6%)
	No	184 (97.4%)
MRI abdomen	Yes	2 (1.1%)
	No	187 (98.9%)

*Patients received solely CT thorax (no CT abdomen)

Number and type of staging diagnostics, findings (insuspicious/suspicious), as well as additional imaging and false positive rates in patients with low-risk EBC are displayed in Table 3 (for patients with Ki-67 < 25%) and Table 4 (for patients with Ki-67 ≥ 25%). In the group of patients with Ki-67 < 25%, chest x-ray showed suspicious results in 2.5%. In patients with Ki-67 ≥ 25%, all chest x-rays were normal. Upper abdominal sonography was abnormal in 2.6% (Ki-67 < 25%) compared to 3.3% (Ki-67 ≥ 25%). Bone scintigraphy was more often suspicious in patients with Ki-67 ≥ 25% (3.8% vs. 11.1%, p = 0.17) whereas CT thorax/abdomen was more often suspicious in patients with Ki-67 < 25% (38.1% vs. 30.0%, p = 0.56). Due to suspicious findings in the initial staging diagnostic, additional imaging was required for 11.8% (n = 12) of patients with Ki-67 < 25% compared to 19.5% (n = 17) patients with Ki-67 ≥ 25% (p = 0.16). The required additional imaging due to suspicious findings are presented in Table 3 and Table 4 respectively. In the first group (Ki-67 < 25%) all suspicious findings were false positive (false positive rate: 7.6%). In the second group (Ki-67 ≥ 25%), the false positive rate was 9.8%. False positive rates did not differ significantly between the two groups (p = 0.55). Distant metastases were detected through additional imaging in one patient (1/87, 1.1%) with Ki-67 ≥ 25%, whereas no metastases were detected in patients with Ki-67 < 25% (p = 0.46). The patient who suffered metastatic disease was diagnosed with asymptomatic bone metastases and had a Ki-67 of 68%.

**Table 3:** Staging diagnostics in n = 102 patients with low-risk breast cancer and a Ki-67 of < 25%

Staging diagnostics in patients with Ki67 < 25%				
	Insuspicious	Suspicious	Additional imaging	False positive rate
Chest x-ray (n = 40, 39.2%)	39 (97.5%)	1 (2.5%)	CT thorax/abdomen (inconspicuous)	2.5%
Upper abdominal sonography (n = 39, 38.2%)	38 (97.4%)	1 (2.6%)	MRI abdomen (inconspicuous)	2.6%
Bone scintigraphy (n = 54, 52.9%)	52 (96.2%)	2 (3.8%)	1 × x-ray femur (inconspicuous) 1 × CT thorax: (inconspicuous)	3.8%
CT thorax and abdomen (n = 21, 20.6%)	13 (61.9%)	8 (38.1%)	6 × CT thorax (inconspicuous) 1 × CT abdomen (inconspicuous) 1 × MRI abdomen (inconspicuous)	38.1%
PET-CT (n = 1, 1.0%)	1 (100%)	0 (0%)	–	–
CT thorax* (n = 3, 2.9%)	3 (100%)	0 (0%)	–	–
Total (n = 158 staging diagnostics)	146 (92.4%)	12 (7.6%)	–	7.6%

*Patients received solely CT thorax (no CT abdomen)

**Table 4:** Staging diagnostics in patients with low-risk breast cancer and a Ki-67 of ≥ 25% (n = 87)

Staging diagnostics in patients with Ki-67 ≥ 25%				
	Insuspicious	Suspicious	Additional imaging	False positive rate
Chest x-ray (n = 36, 41.4%)	36 (100%)	0 (0%)	–	0%
Upper abdominal sonography (n = 30, 34.5%)	29 (96.7%)	1 (3.3%)	Upper abdominal sonography (inconspicious)	3.3%
Bone scintigraphy (n = 63, 72.4%)	56 (88.9%)	7 (11.1%)	1 × MRI thigh (M1) 1 × x-ray tibia (inconspicuous) 2 × x-ray thorax (inconspicuous) 1 × CT pelvis (inconspicuous) 2 × x-ray femur (inconspicuous) 5 × CT abdomen (inconspicuous) 3 × CT thorax (inconspicuous) MRI thoracic spine (inconspicuous) Cerebral MRI (inconspicuous)	9.5%
CT thorax and abdomen (n = 30, 34.5%)	21 (70.0%)	9 (30.0%)	2 × upper abdominal sonography (inconspicuous) 4 × MRI abdomen (inconspicuous) 3 × CT thorax (inconspicuous)	30.0%
PET-CT (n = 2, 2.3%)	2 (100%)	0 (0%)	–	–
CT thorax (n = 2, 2.3%)	2 (100%)	0 (0%)	–	–
Total (n = 163 staging diagnostics)	146 (89.6%)	17 (10.4%)		9.8%

## Discussion

Staging examinations are crucial for patients with newly diagnosed breast cancer to assess prognosis and plan therapy accordingly. However, the incidence of metastasis at the initial diagnosis of EBC with a low-risk profile is low [16–18]. A systematic review of around 15,000 BC patients saw a median prevalence for distant metastases in stage I of 0.2% and stage II of 1.1% [18]. In the present study, we showed a similar result, with only 0.5% of asymptomatic, low-risk EBC (T1-2/N0) suffering distant metastases. Therefore, we can conclude that metastases are generally very rare in asymptomatic EBC. This aligns with previous studies and current guidelines, which suggest that staging diagnostics can be omitted in early stages of breast cancer [11, 12]. In 2012, it has already been recommended by the American Society of Clinical Oncology to avoid staging diagnostics for EBC in order to improve care while reducing costs in healthcare [19]. However, this does not appear to be adopted in clinical practice everywhere [20]. Reasons might be that patients frequently request staging scans and view them as part of "standard care" [21]. Furthermore, there is uncertainty as there might be higher detection rates with new imaging modalities and benefits of staging in more aggressive phenotypes [21]. In the present study, we wanted therefore to investigate whether Ki-67 is a suitable marker for the necessity of staging diagnostics in EBC.

In the present study, we were able to demonstrate that 0% of low-risk EBC patients with Ki-67 < 25% and only 1.1% of low-risk EBC patients with Ki-67 ≥ 25% suffered distant metastases at initial diagnosis. However, 321 different staging diagnostics were performed in total, leading to 29 additional imaging (in 15.3% of patients) due to suspicious findings. False positive rates in our cohorts were 7.6% (Ki-67 < 25%) and 9.8% (Ki-67 ≥ 25%), respectively. This is slightly lower compared to an Australian study, detecting a false positive rate of 15% after initial staging [22]. A Canadian study showed even higher rates of additional staging diagnostics (42%) due to suspicious findings in initial imaging with CT and bone scan in patients with T1-2 and N0-1 breast cancer [23].

Regarding staging diagnostics, previous studies already demonstrated certain limitations. A systematic review of 22 studies found relatively high sensitivity in conventional imaging, while specificity varied (sensitivity/specificity: bone scintigraphy 98.0%/93.5%; chest X-ray 100%/97.9%; liver ultrasound 100%/96.7%; and CT chest/abdomen 100%/93.1%) [18]. However, in a recently published prospective trial involving 410 breast cancer patients, bone scintigraphy showed a lower sensitivity (81.48%), while specificity was 99.09% [24]. In the present study, we observed a false positive rate of 3.8% (Ki-67 < 25%) and 9.5% (Ki-67 ≥ 25%) in bone scintigraphy, which is comparable to the results of Schneider et al. (false positive rate 4.3%) [25]. In our study, chest x-rays revealed only 2.5% suspicious findings in patients with Ki-67 < 25%, and 1.1% of patients required additional imaging due to suspicious upper abdominal sonography. Previously, both chest x-rays [26, 27], and upper abdominal sonography [28] showed diagnostic limitations. Therefore, they have increasingly been replaced by CT scans of the chest and abdomen in recent years. However, CT scans have also shown a 15% false positive rate [22], with no demonstrated change in disease-free survival (DFS) using this staging method [29]. In the present study, CT thorax/abdomen led in 38.1% (Ki-67 < 25%) and 30.0% (Ki-67 ≥ 25%) of performed CT scans to additional imaging or follow-up. Regarding other imaging modalities, benefits of PET-CTs in the context of staging diagnostics for breast cancer were demonstrated in recent years [30–32]. Yet, the routine use of PET-CT is currently not recommended in national and international guidelines, as false negative results can occur, especially with slowly growing metastases or those smaller than 1 cm [11, 12]. In the present study, only 3 patients performed PET-CT as initial staging diagnostics, and all results were normal.

In conclusion we demonstrated that distant metastases are very rare in asymptomatic, low-risk EBC (T1/2, N0). This is in line with previously published studies [16–18]. In the present study, we revealed that with a Ki-67 level below 25%, the likelihood of metastases in low-risk early breast cancer (EBC) is 0%. Consequently, staging tests are unnecessary in these cases. If staging diagnostics are still carried out, this may only lead to unnecessary radiation exposure for the patient during the initial staging diagnostics [33]. Furthermore, it may require additional follow-up diagnostics or histological clarifications due to the high incidence of false positive findings [7]. The psychological stress on patients with a new diagnosis of breast cancer is also significant. Anxiety, depression, uncertainty, and confusion often arise, particularly during the waiting period for test results [34]. Moreover, costs of nonindicated staging (depending on the performed diagnostics) are around $6000 per patient [35]. Additionally, it should be noted that staging tests can delay the initiation of treatment as they require more appointments [36]. The excessive number of unnecessary tests wastes resources that could be better utilized for patients who truly need them.

Limitations of this study are mainly due to the retrospective character. The need for further check-ups and follow-up diagnostics may depend on the respective diagnostician or institutional guidelines. Since this is an analysis of a single institution, no comparison was possible. However, the need for additional staging diagnostics due to false positive results was comparable to the data available in the literature [22, 25]. Moreover, the small sample size makes a definitive statement whether there should be an indication for staging diagnostics in early breast cancer based on Ki-67 levels difficult. Nevertheless, we were able to demonstrate that only one patient with very high Ki-67 in EBC suffered asymptomatic metastases at diagnosis. Whereas 15.3% of all patients required unnecessary additional imaging within 6 months of diagnosis due to “suspicious” findings. Further research is needed to determine if patients with EBC and very high Ki-67 levels could benefit from staging and early detection of metastatic disease.

## Conclusion

In this retrospective, single institution cohort study, we saw a low rate of distant metastases for asymptomatic low-risk EBC patients (T1/2, N0) with occurrence of metastatic disease being 0% for Ki-67 < 25% and 1.1% for Ki-67 ≥ 25%. All in all, staging diagnostics should not be routinely employed in this patient population. In the present study, only patients with a very high Ki-67 level (68%) showed distant metastasis, despite having low-risk EBC. In these cases of high Ki-67 levels staging diagnostics may be discussed with the patient.

## Data Availability

The data presented in this study are available on request from the corresponding author.

## Abbreviations

EBC: Early breast cancer
HER2: Human epidermal growth factor receptor 2
CT: Computed tomography
ER: Estrogen receptor
PR: Progesterone receptor
MRI: Magnetic resonance imaging
OS: Overall survival
